# Clinical Considerations of Ultra-processed Food Addiction Across Weight Classes: an Eating Disorder Treatment and Care Perspective

**DOI:** 10.1007/s40429-022-00411-0

**Published:** 2022-05-02

**Authors:** David Wiss

**Affiliations:** Community Health Sciences Department, Fielding School of Public Health, University of California, Los Angeles, 650 Young Drive South, Los Angeles, CA 90095 USA

**Keywords:** Food addiction, BMI, Eating disorders, Dietary restraint, Weight stigma, Trauma

## Abstract

**Purpose of Review:**

To examine the prevalence rates of ultra-processed food addiction across different weight classes and offer guidelines for diagnosis and treatment. Clinicians are provided with practical considerations in the assessment of ultra-processed food addiction beyond the use of validated instruments.

**Recent Findings:**

The weighted mean prevalence of ultra-processed food addiction is approximately 20% worldwide and varies widely based on the sample. At first glance, there appears a linear relationship between ultra-processed food addiction and BMI class. Further investigation indicates a J-shaped curve with heightened prevalence among the underweight. These findings highlight the need to assess for additional factors that may increase objective or subjective food addiction symptoms including eating disorders, dietary restraint, and other mental health diagnoses.

**Summary:**

While clinical considerations across different weight classes vary, overemphasis on weight status may detract from the clinical utility of the ultra-processed food addiction construct. Considering weight status in conjunction with other psychiatric symptoms helps to better understand the various biopsychosocial mechanisms that influence eating behavior and can inform individualized treatment strategies.

## Introduction

The phenomenon of food addiction (FA) has been mentioned in over 2,500 published articles to date [1•]. Most research studies have operationalized FA using the Yale Food Addiction Scale (YFAS) [2] and more recently YFAS 2.0 [3] based on DSM-5 criteria for substance use disorders (SUDs). FA can be understood as hedonic eating after homeostatic requirements have been met [4, 5] with continued consumption despite negative consequences [6]. Paradoxically, FA symptoms have been reported among underweight individuals who may not be meeting their energy needs [7•], necessitating a closer examination of the FA construct through the lens of eating disorder (ED) psychopathology and related symptoms.

The purpose of this review is to describe the reported prevalence rates of FA across different weight classes and to provide clinical guidelines for diagnosis and treatment, with particular emphasis on the underweight category. The aim is to offer clinicians practical guidelines in the assessment and treatment of FA beyond the use of validated instruments.

There is growing trend toward use of the term “ultra-processed food addiction” (UPFA) emphasizing noteworthy differences from minimally processed foods in their addictive potential [8–12]. Given that the YFAS specifically asks about the consumption of ultra-processed foods with added sugars, salts, and fats, the terms FA and UPFA have been used interchangeably. The UPFA nomenclature may advance the utility and specificity of the FA construct, given that nearly 60% of the calories consumed in the USA come from ultra-processed foods [13]. Henceforth, the term UPFA will be used to describe the phenomenon of food addiction.

While clinical considerations across different weight classes may vary, this review argues that overemphasis on weight status may detract from the clinical utility of the UPFA construct. Weight (and BMI) is often analyzed as an outcome which is influenced by an array of biological, psychological, social, and behavioral causes whereas addictions are defined (and diagnosed) solely by behavioral criteria. Thus, consideration of UPFA as a behavioral health disorder (rather than weight disorder) may provide more insight into clinical treatment strategies.

Several studies have failed to show significant correlations between UPFA and BMI [14–18]; therefore, efforts to address UPFA clinically should not be synonymous with weight loss. Rather, UPFA has been associated with reduced quality of life [19] through mechanisms such as emotion dysregulation [20–22] which can be targeted. Furthermore, UPFA has been associated with altered psychosocial functioning more than with metabolic parameters [18]. Meanwhile, considering one’s weight status in the clinical evaluation of UPFA might be useful to better understand the various biopsychosocial mechanisms that influence eating behavior (including the subjective experience of it).

## Ultra-processed Food Addiction Prevalence

The most recent systematic review and meta-analysis of 272 studies using the YFAS, YFAS 2.0, and derivative scales reported a weighted mean prevalence of UPFA diagnoses at 20% worldwide (95% CI: 18–21%) [1•]. The prevalence of UPFA varies widely based on the sample under study. Clinical samples have higher prevalence than non-clinical samples [1•]. A nationally representative sample from the USA reported UPFA prevalence at 15% [7•], which closely mirrors prevalence rates of other substance-related behaviors in the USA [23]. While the prevalence of UPFA is generally higher in women than men [24–26], these findings are not consistent [7•].

UPFA prevalence estimates are higher in adults compared to children and adolescents (15%; 95% CI: 11–19%) [27], suggesting that symptoms develop over time, much like other addictions. Increased exposure to ultra-processed foods may contribute to early recruitment of brain regions associated with food consumption and choice [9]. A blunted reward experience might increase overconsumption behavior in attempt to reattain the expected reward [28]. Reward-related susceptibility to addictive processes may also be exacerbated by various forms of stress, trauma, and adversity often associated with low socioeconomic status and unhealthful food environments [29].

Recent studies have shown that the COVID-19 pandemic increased the incidence rate of UPFA [30] and associated weight gain from eating behavior attributable to circumstances such as isolation [31]. It has also been documented that COVID-19 led to a surge in EDs [32]. Directionality between UPFA and ED symptoms during the pandemic (and before) remains unclear. A major shortcoming in research linking UPFA to EDs is a failure to disentangle the temporal sequence of disorder onset. This limitation may make the interpretation of rising UPFA prevalence “noisy” because many ED symptoms can increase YFAS scores [33•]. Similarly, UPFA can precede ED behaviors, generating a chicken versus egg conundrum for researchers and clinicians.

## Ultra-processed Food Addiction and Eating Disorders

Clinical samples (such as those with EDs) have higher UPFA prevalence than community-based or representative samples [34]. Specifically, individuals with bulimia nervosa (BN) have the highest prevalence of UPFA (48–95%), followed by binge eating disorder (BED; 55–80%), and anorexia nervosa (AN; 44–70%) [1•, 35•, 36]. Most studies investigating the association between AN and UPFA found that UPFA symptoms are higher among those with AN binge-purge type (AN-BP) compared to the restrictive subtype (AN-R) [37, 38, 39•, 40].

An understanding of the heterogeneity in UPFA presentations necessitates assessment for ED psychopathology, particularly the role of restrained eating and other compensatory behaviors as a cause and/or consequence of UPFA symptoms [33•]. The symptoms of UPFA in under-to-normal weight ranges may represent individuals exhibiting compensatory behaviors (e.g., purging in BN or AN-BP, or over-exercising) which keeps their BMI stable (or decreasing) despite the presence of addiction-like eating (e.g., bingeing, loss of control) commonly associated with weight gain [41•]. It has been suggested that weight control behavior in individuals with BN may dampen reported associations between addiction-like eating and BMI [42, 43].

There is a growing trend toward viewing UPFA and EDs from a transdiagnostic perspective [44–46] particularly in relation to food cravings [47]. The clinical considerations discussed herein are designed to improve our understanding of the UPFA construct in individuals with and without EDs. Equally important, UPFA is frequently seen in subjects without EDs (or other psychiatric disorders) in the general population, which suggests that UPFA is a distinct entity from EDs and other psychiatric illness [48].

## Ultra-processed Food Addiction and Body Mass Index

**Fig. 1:**
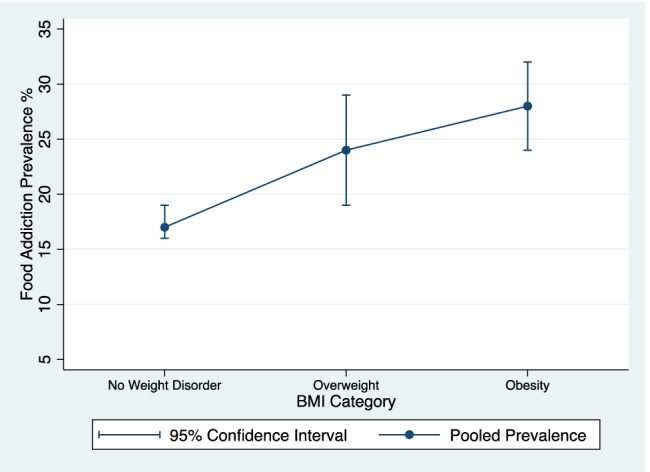
The broadest view of the relationship between BMI and ultra-processed food addiction prevalence from meta-analytic data reported by Praxedes et al. [1•]

Without a nuanced understanding of how UPFA symptoms present clinically in the under and normal weight categories, a linear relationship between UPFA and BMI might be assumed. Figure 1 is adapted from the recent Praxedes et al. meta-analysis [1•] and represents the broadest possible relationship between UPFA and BMI. The pooled prevalence among individuals considered normal weight was 17% (95% CI: 16–19%); among overweight was 24% (95% CI: 19–29%); and among those considered obese was 28% (95% CI: 24–32%). Not enough UPFA studies among the underweight class were available for pooled analysis. At first glance, the relationship between UPFA and BMI appears linear.

Other investigations of UPFA prevalence including individuals with EDs generates J-shaped curves, with the underweight category demonstrating sharply higher UPFA prevalence (12–46%) [7•, 41•, 49•]. A representative USA sample indicated that the prevalence of UPFA is paradoxically highest in the underweight category (46%) [7•]. A representative German sample indicated UPFA prevalence among underweight (15%) as higher than class I obesity (12%) but lower than class II (21%) [49•]. Not surprisingly, prevalence rates differ widely according to sample demographics. It is also possible that food insecurity may increase UPFA symptoms, which may be relevant across BMI classes [50, 51]. Key findings from several recent studies show that obesity cannot be explained solely by UPFA [52–54].

**Fig. 2:**
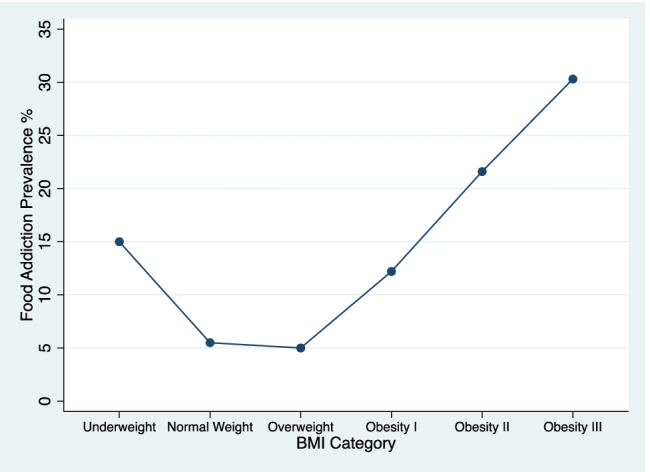
A closer view of the J-shaped relationship between BMI and ultra-processed food addiction prevalence from a nationally representative German sample reported by Hauck et al. [49•]

Data from the German sample [49•] are presented for conceptual purposes in Fig. 2. The prevalence rates in Fig. 2 are not intended for direct comparison to the meta-analytic findings in Fig. 1, but rather to illustrate how a more fine-grained analysis of BMI reveals information that could be overlooked. A non-linear relationship suggests that UPFA is not the only contributor to weight gain and may reflect a distinct phenotype of problematic eating that is not synonymous with obesity [49•]. It is also possible that self-reported UPFA symptoms in the lowest weight class are relics of dieting and caloric deprivation that mimic behavioral indicators of addiction. Examples include eating more than intended due to a violation of dietary rules, or cravings that reflect a homeostatic drive for food from an undernourished state [7•].

In the next section, the literature exploring the non-linear relationship between UPFA and BMI categories are reviewed. Furthermore, mental health considerations are included to assist in developing treatment strategies when positive UPFA screens occur in different BMI categories. Recommendations prioritize ED recovery and are designed to reflect weight-inclusive approaches to managing health [55•]. An argument will be made that UPFA appears primarily related to eating pathology and only secondarily related to body weight [52].

## Ultra-processed Food Addiction and Dietary Restraint

This section reviews often overlooked clinical symptoms among individuals with UPFA who also exhibit high levels of dietary restraint (DR). While binge eating and UPFA are not synonymous, research linking DR to binge eating will be used to conceptualize relationships between UPFA and DR. It is also plausible that dieters may not subjectively binge but rather display other characteristics of addiction-like eating such as social impairment, avoidance, and emotional distress, all of which could increase UPFA severity indicators.

Published data linking UPFA and DR are mixed. Cross-sectional data have linked UPFA scores with higher levels of DR [56] whereas the validation studies of the YFAS and YFAS 2.0 showed no significant correlations between UPFA and DR [3, 57]. Among adolescents in a weight loss program, UPFA was not related to eating restraint [58]. Other investigations of adolescents have shown positive correlations between UPFA and DR [59]. Less is known about the temporal relationship between DR and UPFA symptoms, requiring longitudinal investigations in samples with and without current (or prior) EDs.

While it is well known that efforts to restrain eating often contribute to binge eating, other models suggest that UPFA leads to weight gain and then progress to DR [60]. This pattern is frequently observed among other addictive disorders. For example, repeated alcohol exposure can lead to problematic use which could in turn lead a person to restrict their alcohol use to mitigate these problems. The return to alcohol (i.e., relapse) closely resembles the way some return to highly palatable foods following a period of abstinence [46].

Early research among women with BN revealed that the first binge preceded diet onset in 9–37% of cases [61, 62]. In a cohort study of young women dieting, only 3.5% developed an ED of clinical severity during the 2-year follow-up [63]. A recent review found no experimental support for the DR theories of EDs, suggesting that DR may activate vulnerability factors leading to ED symptoms but only among those already vulnerable [64]. As previously stated, DR may be a consequence of weight gain and the perception of overeating. Therefore, divergent models are needed to better understand directionality between UPFA and DR [60].

Food cravings have been linked to dieting [65], and efforts to restrain eating predicted future overeating during COVID-19 through negative emotions [66]. Data on twins has shown that frequent attempts to lose weight reflect future susceptibility to weight gain [67], but the precise mechanisms remain unclear. A randomized controlled trial using internet- and app-based intervention demonstrated that targeting DR led to greater reductions in binge-eating eating episodes compared with controls [68]. Taken together, it appears that DR may be one contributor to UPFA symptoms such as binge eating and therefore might be a useful target for clinical interventions among individuals meeting criteria for UPFA, especially those with comorbid EDs.

Other research suggests food reward tends to decrease during weight management interventions [69]. A study using caloric restriction for at least 12 weeks reduced food cravings, supporting a deconditioning model [70]. Various dimensions of food cravings are differentially related to outcomes in dieting [65]. Short-term selective food deprivation increases cravings for avoided foods while long-term energy restriction results in craving reductions, indicating a reduction of previously acquired conditioned responses [71].

In summary, DR has been viewed as both the problem and the solution to rising BMIs [72]. Efforts to lose weight or restrict food can make eating pathology worse [73–75]. It is unclear whether efforts to eat differently (emphasizing quality rather than quantity) should be classified as disordered behavior [29] including Orthorexia Nervosa [76], but likely depends on an individual’s psychiatric profile [33•]. Individuals who meet criteria for UPFA and engage in DR may experience varying effects on their body weight, partially depending on whether the restraint is part of a recovery plan, mistargeted (i.e., excessive), inadequately supported, or part of a restrictive ED [29], which can be driven by body dissatisfaction.

## Ultra-processed Food Addiction and Body Image Disturbance

Body image disturbances are known correlates of DR and EDs and are related to quality-of-life measures [77]. Susceptibility to attentional biases toward appearance-related information might be one vulnerability factor in the prolonged persistence of body image disturbances in daily life [78]. Cohort data found that the adolescent drive for thinness continues into adulthood and may predict compulsive eating behavior (reward-based eating) and greater BMI independent of childhood weight [79]. Body image disturbance may persist over the life course and should be evaluated when treating all forms of disordered eating behavior.

Body image disturbance has been independently associated with UPFA symptoms, suggesting that it may be one important factor in the development and/or maintenance of UPFA [80]. While it is likely to be a consequence of UPFA, assessment of body image through validated instruments or by clinical evaluation may be important for developing treatment interventions. For example, if body dissatisfaction drives DR which in turn contributes to UPFA symptoms, targeting body image disturbances might reduce dieting and improve addiction-like eating [81]. While the number of young people with elevated BMIs continues to grow, so has the percentage of youths experiencing body dissatisfaction and weight stigma [82], necessitating incorporation of this discourse into public health campaigns.

### Weight Stigma

Recent conceptual models have suggested that the experience of weight stigma may drive DR and contribute to UPFA symptoms [29]. Among adolescents, UPFA significantly mediated the association between weight-related self-stigma and binge eating [83]. In a longitudinal study of young adults, fear of being stigmatized predicted worsening UPFA symptoms over time [84]. These studies suggest that the experience of weight stigma contributes to UPFA among some individuals. Other research suggests that understanding UPFA broadly reduces weight stigma by minimizing the blame narrative associated with “personal responsibility” [85, 86]. Several authors have proposed that targeting weight stigma at the societal level should be a public health goal [87]. Targeting the internalization of weight bias can be beneficial among those with UPFA, regardless of their weight status.

Anti-obesity messaging may put vulnerable individuals at risk for EDs [88]; a highly undesirable outcome. Meanwhile, evidence-based treatments for obesity can reduce disordered eating behaviors without increasing internalized weight stigma [89]. Divergent views in the field may arise from a failure to separate advocacy from evidence, as well as training/discipline bias. Efforts to find a common ground between the prevention of obesity and EDs are greatly needed [64]. But given that UPFA is not synonymous with obesity, it is imperative that researchers and clinicians understand the potential relationship between weight stigma and UPFA independent of BMI.

## Ultra-processed Food Addiction and Other Mental Health Disorders

A detailed discussion of the associations between relevant mental disorders and UPFA is beyond the scope of this review. Nevertheless, some of the associations between select mental health disorders and UPFA will be summarized to aid clinicians treating UPFA. An 8-step assessment process to discern UPFA from DR using psychiatric symptoms has recently been published [33•]. This process has not been validated but may be useful as a roadmap for conceptualizing UPFA treatment in the context of EDs (emphasizing DR as a proxy for restrictive behaviors associated with EDs).

### Substance Use Disorders

Research linking UPFA to SUDs are mixed. There is some evidence of elevated UPFA prevalence in those with SUDs [90] as well as cross-addiction between food and substances of abuse [91] but these associations were not statistically significant in a recent meta-analysis [92]. Nevertheless, UPFA has been associated with a family history of addiction [20] and brain reward dysfunction [11]. Based on clinical experience, many individuals with severe UPFA do not cross-addict into other substances, partly because highly palatable foods are always available and therefore remain the substance of choice (or no choice).

Impulsivity strongly correlates with problematic substance use [93, 94] and is consistently associated with UPFA [95]. While it has been suggested that a history of SUD might help with clinical implications of UPFA [33•, 46], impulsivity might be a more informative concept. Higher distress-driven impulsivity has been associated with more addiction-like eating behaviors among participants classified as cognitively inflexible [96]. Thus, the presence of any current or previous SUD may be a proxy measure for impulsive behavior when treating UPFA. A family history of SUD should be considered in the assessment [46].

In patients with both UPFA and SUD, targeted treatments that reduce impulsivity and increase self-directedness may improve treatment outcomes [97]. While this may be difficult, evidence of UPFA channeled by trait levels of impulsivity may help explain why addictive-like behaviors can be recalcitrant to change [21]. The associations between UPFA and impulsivity have been shown to associate with more frequent weight fluctuations and higher BMIs [98]. This information may be helpful to clinicians working with disordered eating by reducing stigma surrounding eating pathology, advancing trauma-informed care for addictive disorders [99].

### Trauma and Post-traumatic Stress Disorder (PTSD)

Trauma, particularly early in life, has been associated with increased BMI over the lifespan and UPFA may be one mediating mechanism [43, 100]. The risk for UPFA increases following exposure to early life psychological, sexual, and physical abuse [19, 101]. In fact, all forms of trauma increase risk for UPFA, likely driven by PTSD symptoms [102]. The association between early life adversity and UPFA might be the result of multiple pathways of biological embedding [43], such as reward processing [103, 104] which can heighten risk for SUDs [105] and impulsivity [106].

Associations between early life trauma and EDs are well-described [107, 108] but efforts to investigate the role of UPFA as a mediator, moderator, or outcome are limited. UPFA, as well as emotional eating, have been shown to mediate the relationship between psychological distress and BMI [109]. Based on the available evidence, it appears important to look for signs of trauma and PTSD when designing interventions for UPFA. Considerations include the mechanisms of biological embedding that may impact eating behavior as well as the possibility that some nutrition interventions (e.g., excessive DR) can be triggering for some individuals, particularly those with food-related trauma (e.g., parents who imposed dieting and body shaming [110]).

### Depressive Symptoms

In a systematic review and meta-analysis, the weighted mean correlation between UPFA and depression was 0.46 (95% CI: 0.36–0.55) [92]. This relationship is bidirectional and may be important for psychoeducation in those with UPFA. Ultra-processed food consumption increases the risk of developing depression [111, 112] while “Mediterranean-Style” eating patterns have been associated with reductions in these symptoms [113]. Thus, when depressive symptoms are present, patients with UPFA should be counseled to eat in ways known to reduce these symptoms [114].

There is a U-shaped curve between BMI and depression, where the highest rates occur in underweight and obese categories [115]. It is plausible that associations between underweight and depression stem from undernutrition, although this is not well documented. From clinical experience, counseling efforts focused on mental health outcomes such as depressive symptoms rather than weight are welcomed by most patients (once recent data on weight science have been discussed).

## Clinical Considerations of Ultra-processed Food Addiction Across Weight Classes

In this final section, clinical considerations for UPFA are broken down by weight classes, with emphasis on the under and normal weight categories. Differentiation by weight status does not imply that UPFA treatment should be determined by BMI alone, but rather that BMI might influence intervention priorities. It is important to distinguish UPFA from restrictive EDs before UPFA-related treatment is implemented.

Recommendations are generated from available evidence and the clinical experience of the author which have not been validated. Systematic reviews of interventions for UPFA have recently been published [116, 117]. Ketogenic diets used in the treatment of binge eating and UPFA [118, 119] are controversial and are not discussed here but may have utility in select cases.

### Underweight

The heightened prevalence of UPFA among those in the underweight BMI category and its treatment has not been well described. When other comorbid conditions have been identified, it is useful to explore the items on the YFAS that specifically contribute to elevated UPFA scores. An interview-style assessment is helpful to ascertain if these individuals engage in objective or subjective overeating [7•]. Other questions such as changing social, occupational, or recreational activities, or continuing to eat despite physical or psychological problems may reflect subjective experiences, especially when considering ultra-processed foods that often elicit moral judgments about these foods [34].

The six clinical considerations below are derived from an expanded 8-step process designed to help separate overlapping signs and symptoms of UPFA and DR [33•] but did not consider differences across BMI categories. When UPFA is detected among the underweight class:

1. Rule out food insecurity or other forms of socioeconomic disadvantage or deprivation that might be elevating UPFA symptoms.
2. Assess for the presence of AN, particularly AN-BP. If present, the current evidence-based treatment includes weight restoration [120]. Obsessive–compulsive disorders and anxiety frequently occur when AN is present [121, 122]. In patients with AN-R, a high YFAS score often reflects fear of overeating, bingeing, or loss of control rather than the actual behavior [123]. This concept is supported by evidence of AN patients reporting greater UPFA symptomatology while being able to successfully regulate their food cravings [124]. Recent research has suggested there are two distinct phenotypes among those with co-occurring AN and UPFA: (1) restriction that drives perceived or actual UPFA symptoms; and (2) genuine UPFA that is premorbid to AN, where a heightened reward response to ultra-processed foods leads to restriction or fasting [39•]. If the latter is present, nutrition interventions aimed at reduced exposure to ultra-processed foods while simultaneously pursuing weight restoration may be clinically appropriate. It may also be useful to discuss the pros and cons of engaging in a 12-Step program for compulsive eating, as these groups can be attractive to those feeling misunderstood by clinicians but can reinforce restrictive ED symptoms in some cases [125].
3. It is important to assess for DR and other compensatory behaviors (e.g., excessive exercise) in the absence of clinically significant ED that may be driving weight loss. If these behaviors are driven by body dissatisfaction, therapeutic interventions aimed at promoting positive body image can be helpful [126]. When present, it is important to address restrained eating and the relentless pursuit of thinness as risk factors for EDs and potential predictors of compulsive eating and rebound weight gain later in life [79]. Investigating weight history is informative to better understand metabolic and psychological concerns associated with weight-cycling [127]. After weight restoration, the goal is weight stability. Outcomes are improved when a registered dietitian nutritionist specialist is involved [128].
4. It is important to assess for the presence of current SUD which may drive malnutrition [129]. When present, evidence-informed recommendations should address the specific substances used [130] while abstinence or harm-reduction strategies are being pursued.
5. Determine if trauma symptoms or trauma history is present and provide general psychoeducation that links adversity to food and body issues as one part of an established evidence-based trauma treatment [131]. Overlooking trauma may contribute to poor treatment outcomes because unresolved adversity may be considered a fundamental cause of all downstream psychopathology [132].
6. Evaluate for depressive symptoms and if present, consider the possibility that undernutrition, body image disturbance, or pathological restrained eating is contributing to this outcome and target all three potential vulnerability factors. In some cases, anti-depressant medications may be indicated.

### Normal Weight

The normal weight class has the lowest UPFA prevalence, but there are important clinical considerations which aim to distinguish contributions from EDs such as BN and BED. Much like the underweight category, investigating the specific YFAS questions which lead to a positive UPFA screen might help clarify subjective versus objective overeating. Once this assessment has been made, continue to the steps outlined for the overweight/obese class. Treatment for UPFA in the normal weight class includes:

1. Investigate BN and BED and address associated behaviors such as bingeing, purging, and restricting with the goal of reducing or eliminating these behaviors. Abstinence from purging is an important goal but may take time, particularly in outpatient settings. Identifying “trigger foods” with the goal of abstinence is controversial among ED professionals. It can be helpful to determine the order of UPFA or ED manifestation. If the ED came first, traditional ED treatment which incorporates these foods might be the safest course. One of the goals of normalizing these foods is to disrupt the cognitive distortion of good versus bad foods. If UPFA appeared first, consider excluding specific foods that contribute to bingeing, but only after other clinical considerations have been identified.
2. Same as #3 for the underweight class. Discussion of weight stigma in society and internalized weight bias can be helpful. A common goal is to avoid the relentless pursuit of thinness by stressing consumption of an appropriate amount of food from all macronutrient categories at meals and throughout the day.
3. The clinician should assess for comorbidities such as SUDs which may be contributing to addiction-like eating. If present, use substance-specific protocols [130] while pursuing abstinence or harm-reduction strategies. The temporal sequence between disorders of food and other substances should be investigated. If UPFA symptoms clearly preceded other substance use, this may favor moving toward abstinence from addictive foods, after other clinical considerations have been made.
4. Same as #5 for the underweight class.
5. Same as #6 for the underweight class with additional consideration that excessive reward activation may contribute to this outcome, aiming to reduce addiction-like eating gradually over time.

### Overweight/Obesity

All weight categories above normal have been merged to emphasize that UPFA recovery can be pursued independent of weight status. Clinicians and patients may assume that weight loss is the key outcome of UPFA recovery, but this assumption detracts from important quality of life outcomes which should be prioritized. The likelihood of transitioning from an obese classification to normal weight over a 9-year period is less than 1% [133]. For this reason, other goals such as reducing disordered eating, other addictive behaviors, trauma symptoms, and depressive symptoms should be prioritized. When patients lose weight during UPFA treatment, it is often the result of better nutrition and lifestyle habits, as well as improved stress management. One way to deemphasize weight in UPFA treatment is to avoid weighing people throughout treatment, as well as discourage them from weighing themselves.

If UPFA is detected among the overweight/obese class, the steps outlined for normal weight should be pursued first. Once these considerations have been made, UPFA may exist as a separate entity from an ED or other diagnosis. When UPFA has become the primary target for recovery:

1. Create a meal strategy that is consistent in nutrients with positive sensory experiences with food. Avoiding hunger and gaps in nutrients are important in creating a consistent (yet varied) experience with food to reduce hedonic overeating [134•]. For example, three meals plus one or two snacks containing all the macronutrients at meals. Deficiencies in fiber, protein, and fat might contribute to hunger which could increase the risk for hedonic eating. Meals and snacks consumed throughout the day should include food that is hot, cold, crunchy, creamy, savory, and sweet (i.e., fruit). Over-restricting caloric intake is not recommended, as the goal is to reduce symptoms of UPFA [134•]. Cravings should diminish over time and emphasizing patience is critical.
2. Switching the goal from weight loss to improved mental health quality of life can reduce the perception of weight stigma. Addressing internalized weight bias might reduce feelings of stress and adversity that in turn make improvements in eating more accessible [29]. Treatment professionals should discuss the issue of fat shaming in society [135].
3. Move toward a low glycemic carbohydrate diet (e.g., beans and high-fiber whole grains) given that high glycemic carbohydrates (e.g., refined grains and added sugars) can interact with mesolimbic dopamine systems that heighten food cravings and may lead to loss of control [45, 136]. Identifying individual trigger foods that should be avoided can be helpful. Usually, these foods are a combination of high fat and refined carbohydrates [12]. Foods to avoid can be modified throughout the treatment and recovery process using trial-and-error as well as exposure-based therapies.
4. Emphasize cooking meals at home rather than relying on purchasing prepared food. Not only does this improve the ingredients used in preparation but can foster a connection with food (and future-directed thinking) that reduces impulsive food choices (author anecdote). While improving the home food environment can be helpful, many individuals live with family members who consume their trigger foods, making recovery difficult. However, recovery can be enhanced by pursuing steps five through seven below.
5. Target impulsivity and habitual patterns of eating by focusing on the neurobehavioral correlates of self-regulation through practices such as mindfulness-based techniques [137, 138•]. Interventions targeted at improving executive function including episodic future thinking, meditation, and exercise can be helpful to support recovery of dysfunctional reward-related processes [139].
6. Provide evidenced-based treatments for improving emotional regulation [140–142].
7. Encourage resilience through social support. The perception of lack of social support has been associated with UPFA [143]. Targeting positive social connections is beneficial for other forms of addiction recovery and should not be overlooked in disorders of eating [45]. 12-Step programs such as Overeaters Anonymous is one well-established example [125]. The addiction framework may reduce perceptions of personal failure [138•] which may be ameliorated through engagement with a like-minded community.

## Conclusions

It is critical to understand the social, psychological, cognitive, behavioral, and physiological factors in the UPFA construct [5]. This includes EDs, DR, body image disturbance, weight stigma, and the presence of other psychiatric comorbidities including SUDs, trauma/PTSD, depressive symptoms, and others. Providers should screen for associated conditions before designing clinical interventions for UPFA. Clinicians should address their own conscious or subconscious biases often when working in mental and behavioral health settings.

Individuals with UPFA require additional psychosocial support along with lifestyle modification. If not executed through an ED-informed and weight-neutral lens, interventions for UPFA can be dangerous, increasing the risk for clinically significant EDs among vulnerable groups. In addition to considering the presence of other comorbidities, investigating which individual questions on the YFAS contribute to a positive screen can be informative. The temporal sequence of disorder onset can also help with case conceptualization but is only one of many puzzle pieces. It may take several sessions including some trial-and-error and discussion of the pros versus cons of different approaches before treatment trajectories become clear.

If a genuine UPFA exists, the steps outlined in this review can be used for interventions that include abstinence or harm reduction, with the goal of improving mental health quality of life rather than weight modification. While weight loss frequently occurs in UPFA-informed interventions, providers should emphasize weight-neutral language and weight-inclusive approaches to reduce weight stigma and provide body-positive affirming care. One goal of UPFA treatment should be to end weight-cycling and create consistency with food. Stratifying by BMI class can aid in prioritizing treatment goals but should not supersede other clinical considerations. Clinical trials are needed to improve treatment outcomes for UPFA and would benefit from collaboration (rather than competition) from ED professionals. Cross-disciplinary efforts from all biopsychosocial domains will be critical to advance the field of food addiction.
